# An Electromagnetic Low-Frequency Flextensional Transducer for Acoustic Logging

**DOI:** 10.3390/s25247481

**Published:** 2025-12-09

**Authors:** Baiyong Men, Huijun Yu, Mingming Jiang, Junqiang Lu, Xiaohua Che, Shizhen Ke

**Affiliations:** 1 China University of Petroleum, Beijing 102249, China; 2 National United Engineering Laboratory for Biomedical Material Modification, Dezhou 251100, China; 3 Northern Information Control Research Institute Group Co., Ltd., Nanjing 211153, China

**Keywords:** acoustic logging, low-frequency flextensional transducer, magnetic circuit, finite element simulation

## Abstract

Low-frequency acoustic logging transducers are pivotal to far-acoustic imaging logging technology and permeability logging technology. This study presents a monopole acoustic transducer driven by electromagnetic force, exploiting the low-frequency vibration characteristics of a flextensional shell. Finite element simulations were employed to evaluate multiple magnetic circuit configurations under dimensional constraints typical of logging tools. An inner magnet circuit was selected and optimized through parametric analysis. Concurrently, the vibration shell was designed and simulated under borehole conditions, accompanied by the development of a dedicated excitation circuit. The fabricated prototype (64 mm outer diameter, 154 mm height, 100 mm shell height) demonstrated operation frequency at 1300 Hz with a sound pressure level of approximately 150 dB and uniform circumferential radiation, satisfying the requirements of logging applications.

## 1. Introduction

Acoustic logging, a core geophysical logging technique, relies on the generation of controlled elastic waves within the borehole and the measurement of their propagation through the adjacent rock formations. A downhole tool, specifically deployed within the wellbore for acoustic logging applications, is equipped with a transducer that emits short-duration acoustic pulses. These acoustic pulses induce three distinct wave modes—compressional (P-wave), shear (S-wave), and Stoneley waves in both the borehole fluid and the surrounding formation. One or more receivers, positioned at fixed axial distances (spacing) from the transmitter, detect the arriving acoustic energy. Monopole transducer configurations primarily excite P-waves and, under certain conditions, can also induce S-waves. Dipole transmitters are specifically designed to efficiently generate and detect flexural waves, which reliably yield shear slowness even in slow formations. Acoustic logging is a widely utilized geophysical well logging technique that can be used to calculate rock elastic parameters (Young’s modulus, bulk modulus, and Poisson’s ratio), estimate maximum and minimum in situ stresses, calculate porosity, and evaluate reservoir productivity [1,2,3,4,5,6]. With the increasing demand for petroleum, natural gas, and mineral exploration, acoustic logging has been extended to investigate geological structures and targets several meters to tens of meters away from the borehole. Far-acoustic imaging logging techniques have therefore been proposed [7]. The far-acoustic imaging technology based on a dipole source operates at low frequency, with a detection range of up to 80 m, but has poor azimuth resolution and suffers from 180° ambiguity [8,9,10,11,12]. The far-acoustic imaging technology based on phased array receiving technology with a monopole source can achieve an azimuth resolution of 22.5°. However, due to the high operating frequency (8–18 kHz), the detection distance does not exceed 40 m [13,14,15,16,17]. Since acoustic attenuation in formations increases exponentially with frequency, reducing the frequency significantly extends the detection distance. Prior studies indicate that when the source frequency is below 3 kHz, Stoneley wave attenuation is highly sensitive to formation permeability, which is the basis of permeability logging technology [18,19,20]. Conventional monopole transducers based on piezoelectric ceramics generally operate well above 8 kHz, which restricts the detection distance of the far-acoustic imaging logging techniques and limits the generation of pure Stoneley waves, thereby reducing the measurement accuracy of formation permeability [21,22]. Thus, a low-frequency monopole transducer is critical to advancing far-acoustic imaging logging and Stoneley wave permeability inversion.

The flextensional transducer concept originated from Hayes’s patent in 1936. These transducers amplify the longitudinal deformation of a driving element into the lateral flextensional motion of a shell, radiating acoustic energy efficiently [23,24,25,26,27,28,29,30]. Conventional piezoelectric-based monopole transducers employed in acoustic logging exhibit resonance frequencies in excess of 8 kHz. Due to borehole dimensional constraints, decreasing the resonance frequency by increasing the ceramic size is impractical. FlextensionalFlexural transducers are widely used in underwater acoustics but are not typically optimized for confined downhole environments. Here, we propose a low-frequency electromagnetic flextensional monopole transducer driven by a coil, which employs the shell’s low-frequency vibration while enabling efficient frequency tuning via control of the excitation current.

This research first determined the key parameters of the magnetic circuit and flextensional shell through simulation analysis. Subsequently, a transducer was fabricated, and a drive circuit was designed accordingly. Finally, experiments were conducted to verify the effectiveness of the design. This paper mainly consists of four parts: I. Design of the transducer; II. Simulation of magnetic circuits and flextensional shells; III. Excitation circuit design and experimental validation; IV. Conclusions.

## 2. Design of the Transducer

**Figure 1:**
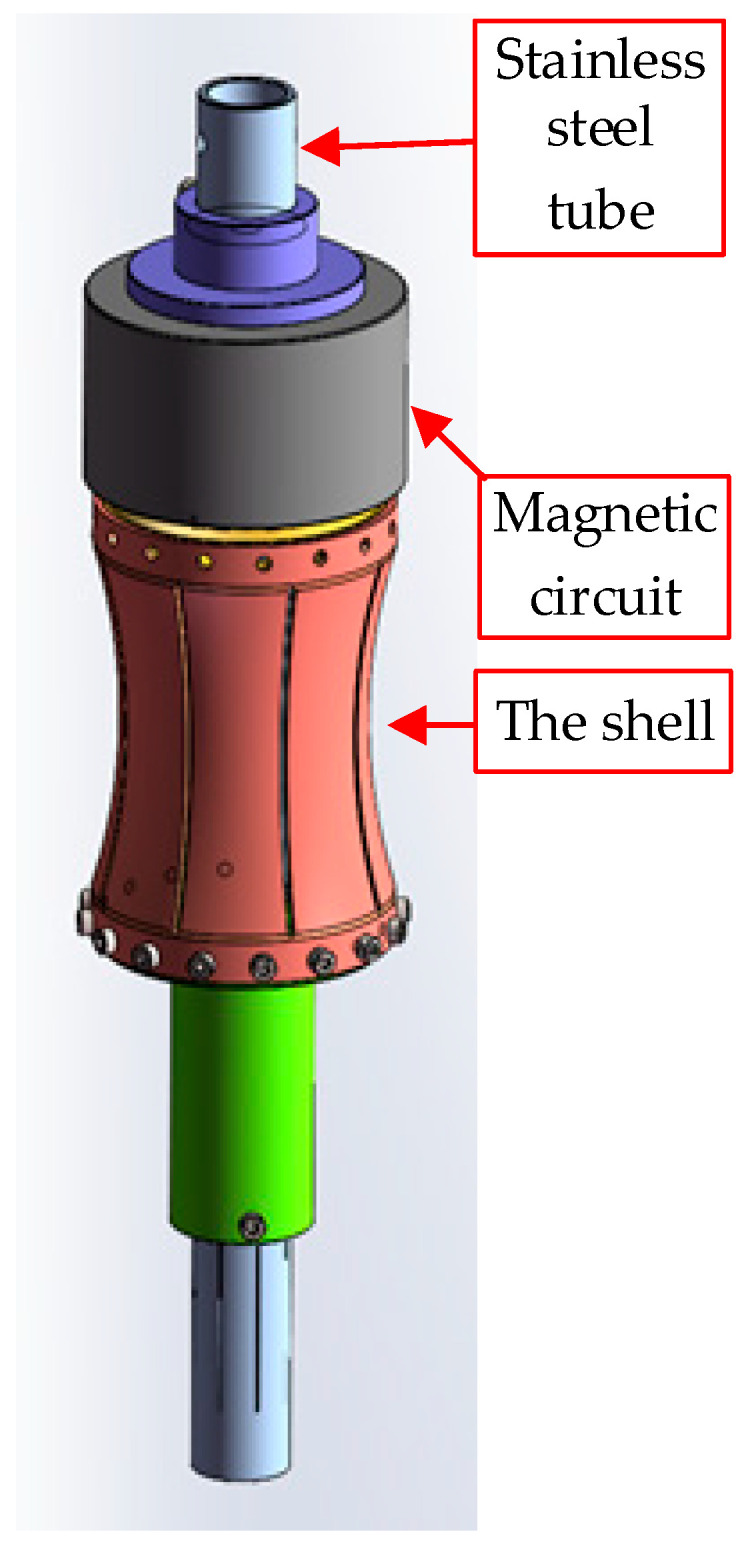
Three-dimensional model of the transducer.

Considering the structure of logging tools, the proposed electromagnetic flextensional monopole transducer adopts a cylindrical form composed of a magnetic circuit, a coil, and a flextensional shell. A three-dimensional model diagram of the transducer is shown in Figure 1. The magnetic circuit generates a static magnetic field in the air gap. The coil is installed at the upper end of the flextensional shell and placed in the air gap. The coil converts alternating current to oscillating Lorentz forces. The flextensional shell transforms mechanical vibrations into acoustic waves. When alternating current flows through the coil in the static magnetic gap, the coil is subjected to oscillating Lorentz forces, which drive the flextensional shell to generate acoustic radiation. The resonant frequency of the transducer is primarily determined by the shell’s mechanical geometry, which can be designed to resonate at low frequencies. By matching the excitation frequency to the shell’s natural frequency, maximum output can be obtained. Frequency regulation can be conveniently achieved by adjusting the driving circuit parameters.

Firstly, the magnetic circuit and shell were analyzed by finite element software COMSOL Multiphysics Version 5.2 [31,32,33,34]. Several magnetic circuit topologies were investigated with different permanent magnet and yoke configurations. Subsequently, frequency-domain and mode-domain analyses were carried out on the shell to assess resonance behaviors and acoustic radiation properties. Based on these results, the optimal configuration for downhole conditions was determined. Then, the excitation circuit was designed based on sinusoidal Pulse Width Modulation (PWM) principles using a Field Programmable Gate Array (FPGA) for precise frequency regulation.

## 3. Simulation of Magnetic Circuit and Flextensional Shell

### 3.1. Magnetic Circuit Simulation

**Figure 2:**
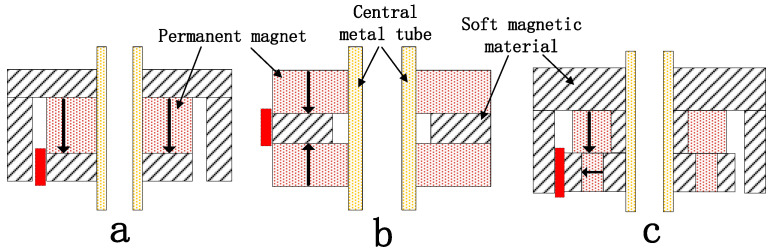
Three types of magnetic circuit models.

The magnetic circuit is the core driver of the transducer, directly determining output performance. Because coil turns and current amplitude are limited by hardware constraints, optimization of the magnetic circuit becomes crucial. Three magnetic circuit models were proposed for confined downhole environments: (a) inner magnet circuit, (b) dual-magnet circuit, and (c) hybrid radial-axial magnet circuit. Figure 2 shows the cross-sectional diagrams of the three types of magnetic circuit models. The red area is the air gap. In type (a), the permanent magnet is situated at the center, with soft magnetic materials disposed above and below it. The upper soft magnetic component exhibits an L-shaped cross-section, which, in conjunction with the lower soft magnetic material, forms an air gap within the red region. In type (b), the soft magnetic material is positioned at the center, with permanent magnets arranged on both the upper and lower sides. The two permanent magnets have opposite magnetization directions, creating an air gap (as shown in the red region) on the outer side of the soft magnetic material. In type (c), the upper section features an L-shaped soft magnetic component in cross-section. The central region contains a vertically magnetized permanent magnet. The lower assembly integrates a permanent magnet with soft magnetic materials, where the permanent magnet is centrally positioned with soft magnetic elements on both sides, exhibiting horizontal magnetization. This arrangement forms an air gap (as indicated by the red region) between the upper soft magnetic component and the outer soft magnetic elements of the lower assembly. The permanent magnet is a temperature-enhanced neodymium iron boron magnet with a remanence of 1.4 T and a maximum operating temperature of 180 °C. The soft magnetic material is electromagnetic iron, which has a relative permeability of 2000 and remains nearly constant below 350 °C.

**Figure 3:**
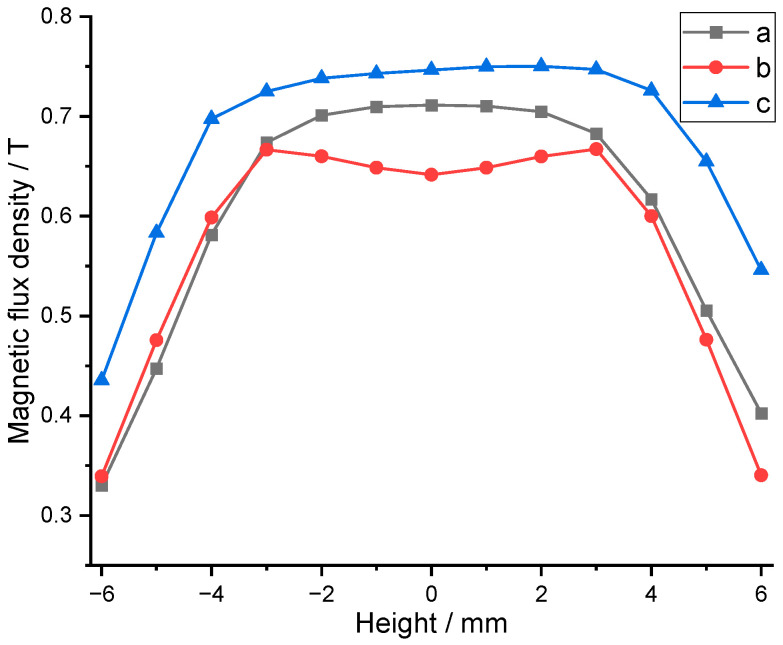
Comparison of the air gap flux density of the three types of magnetic circuit.

Figure 3 presents a comparison diagram of the air gap flux density of the three types of magnetic circuits. The vertical axis represents magnetic flux density, and the horizontal axis represents the positional coordinate of the air gap, with 0 mm corresponding to the midpoint of the air gap. Finite element simulations demonstrated that the hybrid radial-axial magnet circuit exhibited the highest flux density in the air gap, with the inner magnet circuit following closely. However, considering the practical processing costs and manufacturing challenges of permanent magnets and soft magnetic materials, the transducer ultimately adopted an inner magnet circuit design.

**Figure 4:**
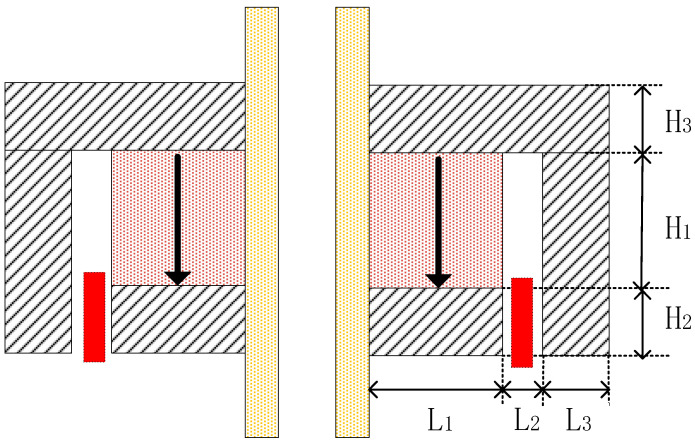
Cross-sectional view of the inner magnet circuit with mechanical dimensions.

**Figure 5:**
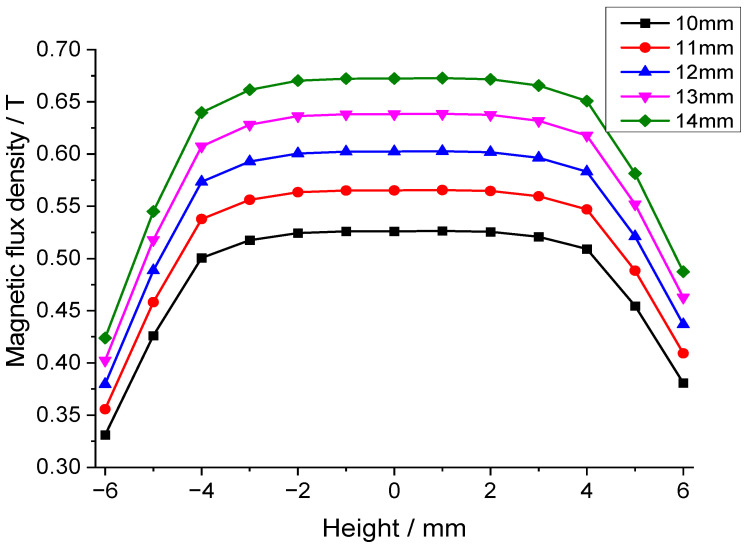
Effect of permanent magnet width on the air gap flux density.

**Figure 6:**
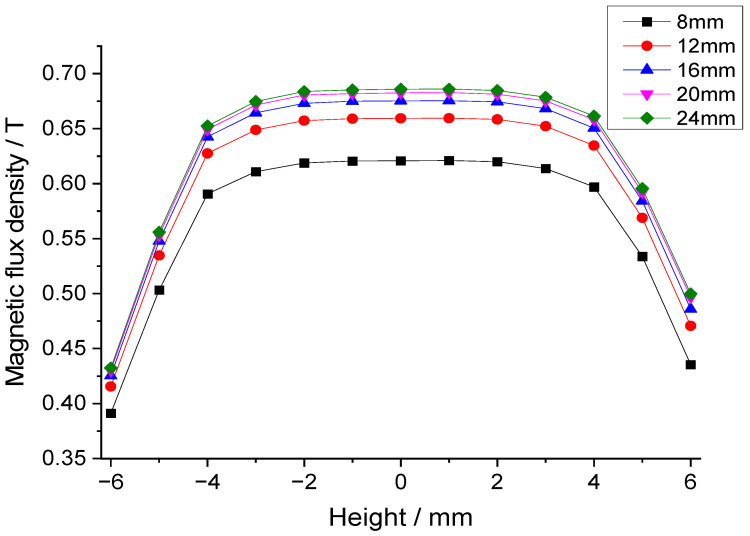
Effect of permanent magnet height on the air gap flux density.

Figure 4 is the cross-sectional view of the inner magnet circuit with mechanical dimensions. Through finite element simulation analysis, the influence of dimensional changes in permanent magnets and soft magnetic components on the flux density in the air gap of the magnetic circuit was investigated. The effect of permanent magnet width (L_1_) on air gap flux density is shown in Figure 5, which indicates that increasing the permanent magnet width significantly increases the flux density. Therefore, the maximum permanent magnet width (14 mm) within the space constraints under the borehole was chosen. Figure 6 is a chart of the effect of permanent magnet height (H_1_) on air gap flux density. As shown in Figure 6, when the permanent magnet height is relatively small, increasing its height rapidly enhances the air gap flux density. However, once the magnet height reaches a threshold, further increases have a negligible effect on the air gap flux density. Consequently, a height of 20 mm for the permanent magnet was determined to be the optimal trade-off for balancing the magnetic flux density against the dimensional constraints. Additionally, simulation results indicate that increasing the volume of the soft magnetic components alleviates local magnetic saturation, thereby enhancing the flux density in the magnetic air gap.

**Figure 7:**
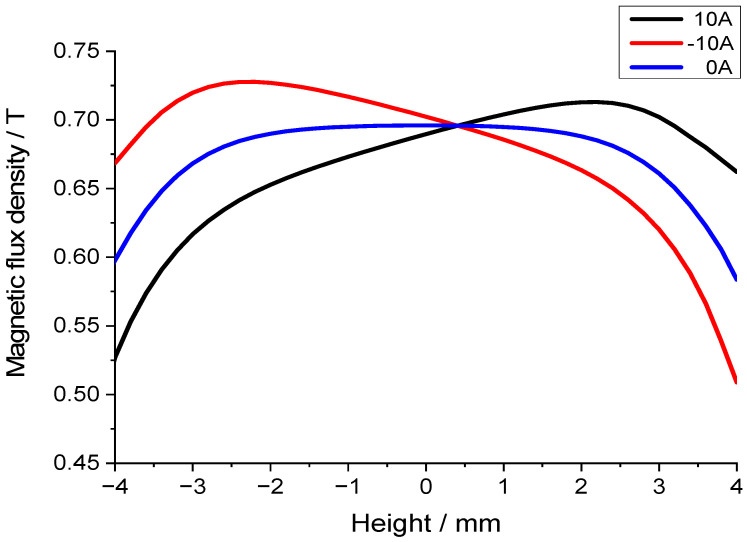
Effect of coil current on the air gap flux density.

The coil of the transducer is positioned within the air gap. When alternating current energizes the coil, the resulting electromagnetic field interacts with the magnetic field in the air gap, thereby modulating its distribution. In practical operation, sinusoidal AC excitation generates a coil current comprising positive and negative half-cycles. This can be emulated in simulations by applying DC currents of opposing polarities. Figure 7 is a comparison chart of the effect of coil current on the air gap flux density. Current through the coil modifies flux distribution but has a negligible effect on the mean flux density.

**Figure 8:**
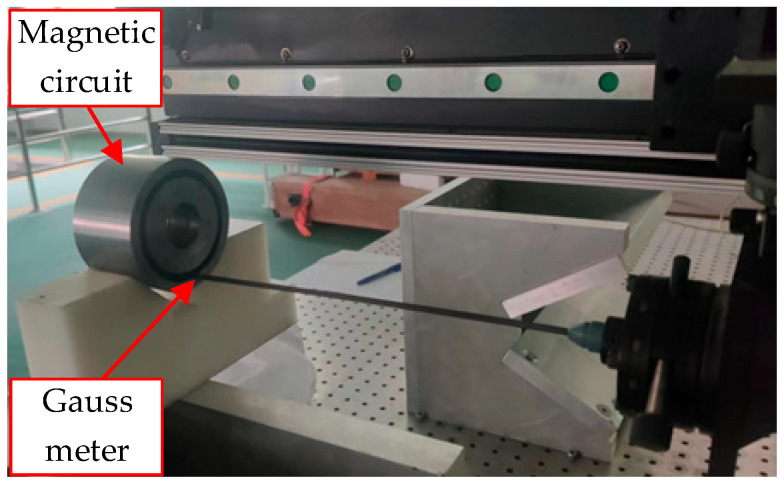
Diagram of the air gap flux density measurement.

**Figure 9:**
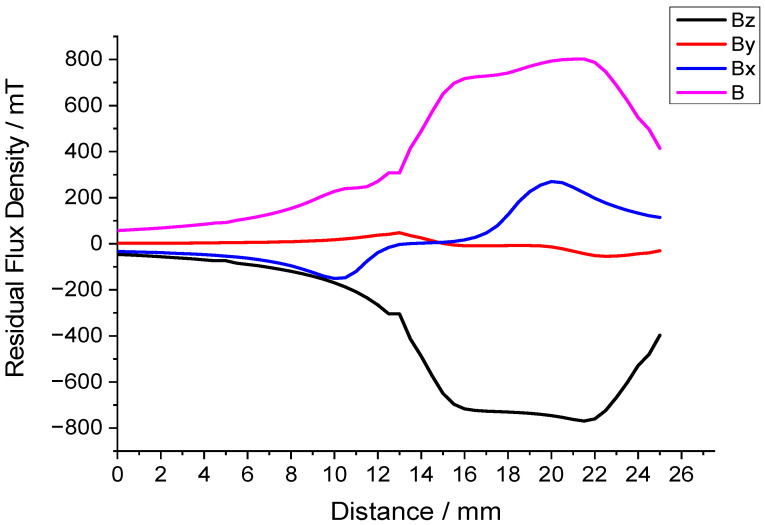
Chart of magnetic flux density variation in the air gap.

After the simulation analysis, the magnetic circuit was constructed. Measurements with a Gauss meter confirmed an average magnetic flux density of around 0.7 T in the air gap, validating the simulation results. Figure 8 is an experimental diagram of the air gap flux density measurement, and Figure 9 is a graph of the magnetic flux density variation in the air gap. The 19 mm position is the midpoint of the air gap.

### 3.2. Flextensional Shell Simulation

**Figure 10:**
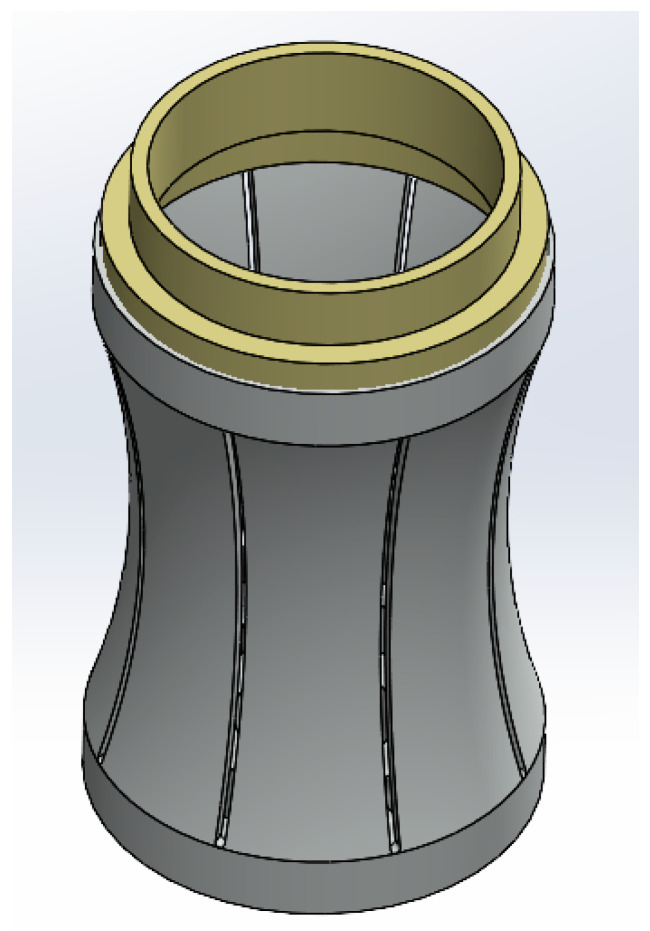
Three-dimensional structure of the vibrating shell.

**Figure 11:**
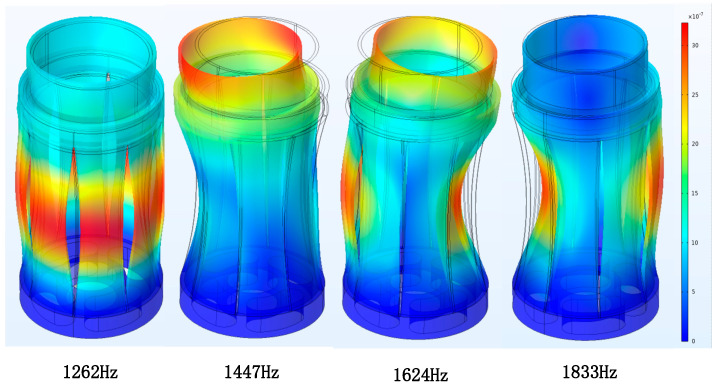
First four vibration mode shapes of the shell.

**Figure 12:**
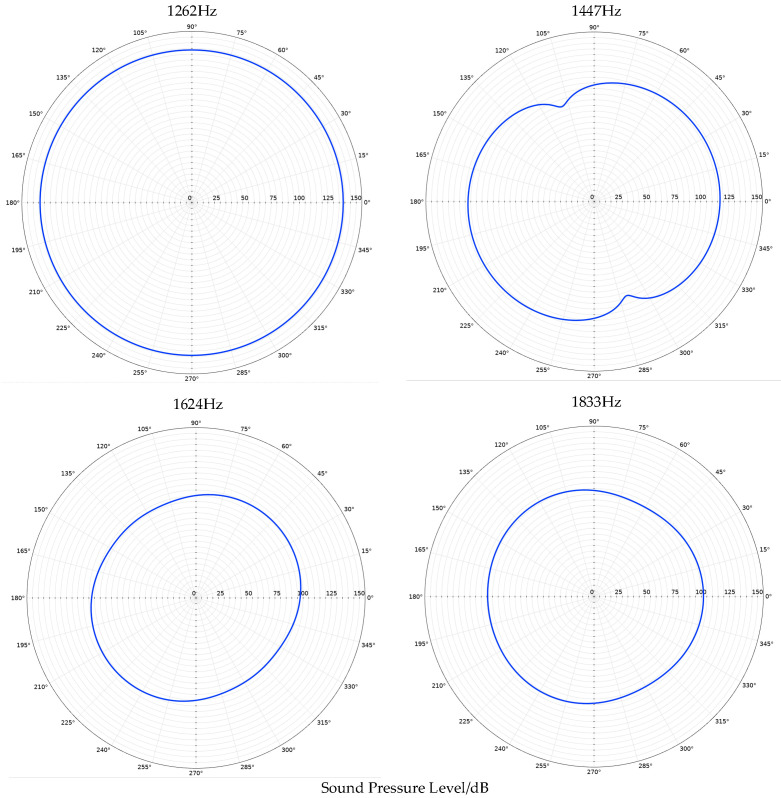
Acoustic pressure produced by the first four vibration modes of the shell at 1 m.

Among the seven types of flextensional transducers, only the concave-shell type (Class I) matched the monopole geometry characteristics. A three-dimensional shell model was established with an end cap (PEEK) and eight aluminum-alloy Flextensional plates, as shown in Figure 10. The finite element simulations’ result revealed that the shell model has multiple mechanical vibration modes. The first four vibration shapes of the shell are shown in Figure 11, and the radiated acoustic pressure produced by the first four vibration modes of the shell at 1 m is presented in Figure 12.

At the first-order mode (1262 Hz), all plates deformed uniformly, producing maximum monopole-like radiation with circumferential uniformity. Higher modes exhibited more complex deformation and non-uniform sound fields. Therefore, the target excitation frequency was set near 1262 Hz.

## 4. Excitation Circuit Design and Experimental Validation

An excitation circuit was designed and constructed to validate the novel transducer.

### 4.1. Excitation Circuit Design

The electromagnetic driving principle relies on high current excitation. To address this requirement, a tunable sinusoidal excitation circuit was designed, capable of delivering a peak current of up to 40 A. When the excitation frequency matches the resonance frequency of the shell, the maximum acoustic output is achieved.

**Figure 13:**
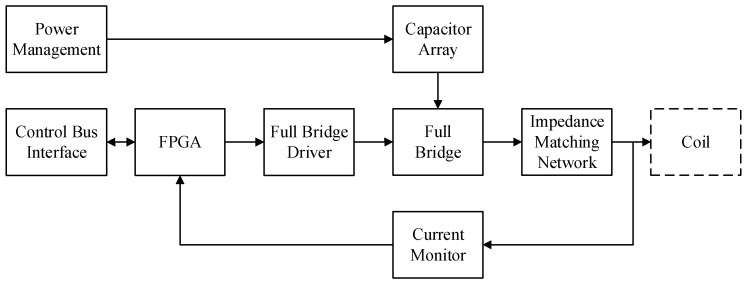
Excitation circuit structure diagram.

**Figure 14:**
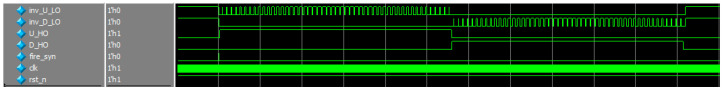
PWM control signal simulation diagram.

Figure 13 presents the schematic diagram of the circuit. The circuit employs a FPGA as its main controller, integrating key functional modules including a power supply, full-bridge driver, impedance matching, and current sensing. Specifically, the FPGA generates Pulse-Width Modulation (PWM) signals, which are fed through the full-bridge circuit to synthesize a sinusoidal current. Additionally, the integration of Direct Digital Synthesis (DDS) technology enables flexible and precise control of excitation frequency. The simulated waveform of the PWM control signal is illustrated in Figure 14.

### 4.2. Experimental Validation

**Figure 15:**
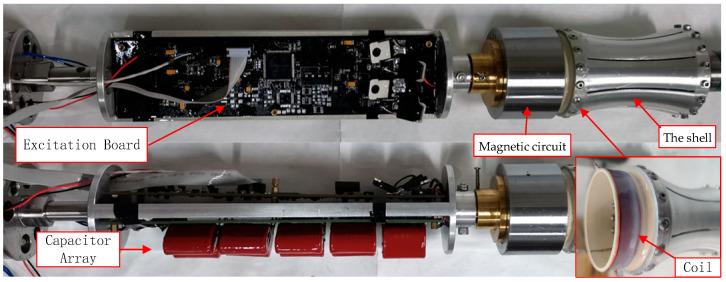
A prototype of the flextensional transducer.

**Figure 16:**
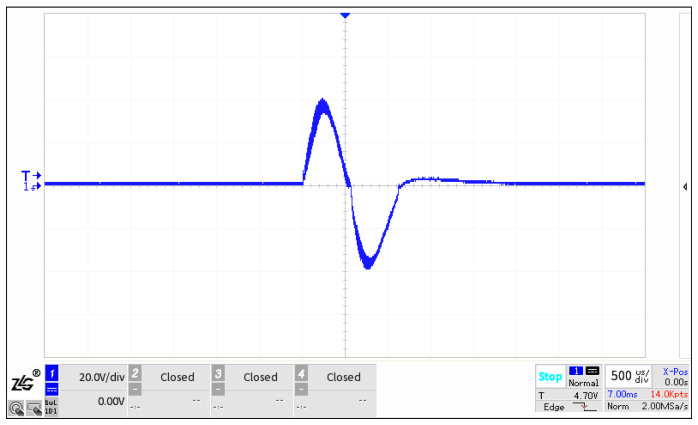
A waveform of excitation current for the transducer.

A prototype of the fabricated flextensional transducer is presented in Figure 15. It consists of a magnetic circuit, shell, excitation circuit, and mechanical connectors, which are all encapsulated in a silicone-oil bladder. The outer diameter and the overall height of the prototype are 64 mm and 154 mm, respectively. The flextensional shell height is 100 mm. The waveform of the excitation current for the transducer is shown in Figure 16. It illustrates that the peak excitation current reaches 40 A. The measurement equipment for the excitation current includes a Tektronix TCP0150 current probe, a Tektronix TCP305A Current Probe Amplifier, and an oscilloscope, with the 1:1 current–voltage conversion ratio.

The experimental tests comprised two phases. In the first phase, frequency sweep excitation was employed to ascertain the transducer’s resonant frequency. Due to discrepancies between the actual material properties of the transducer and those in the simulation model, coupled with manufacturing tolerances, the actual resonant frequency may deviate from the simulated value. Thus, frequency sweep excitation is essential for the accurate ascertainment of the resonant frequency. In the second phase, following confirmation of the resonant frequency, the directivity and sound pressure level (SPL) of the radiated acoustic field were quantified.

**Figure 17:**
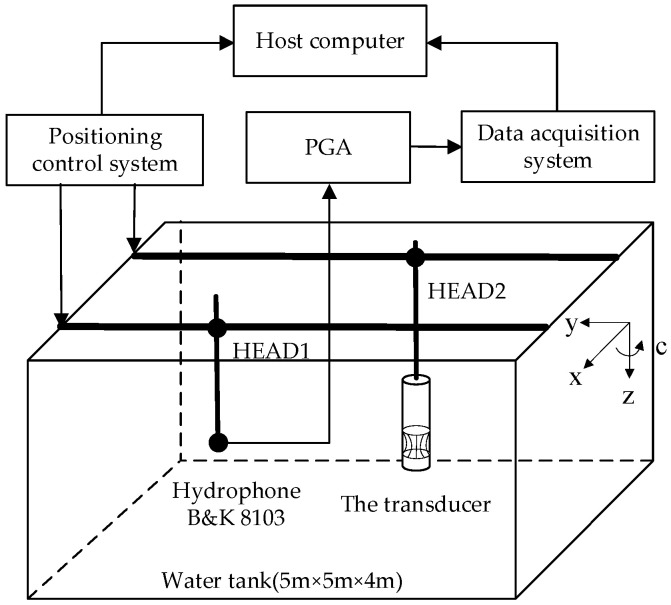
Schematic diagram of the experimental setup.

The acoustic performance was characterized in a 5.0 × 5.0 × 4.0 m water tank. The experimental system consists of a host computer, a positioning control system, a home-built multichannel acquisition system, and a programmable gain amplifier (PGA), as shown in Figure 17. The positioning control system is equipped with two moving heads, each of which has four degrees of freedom, namely translations along the x-, y-, and z-axes as well as rotation in the circumferential direction (c-direction). The hydrophone (Brüel & Kjær 8103) was mounted at the bottom end of HEAD1, while the transducer was installed at the bottom end of HEAD2. The positioning system is connected to the host computer via Ethernet to conveniently control the movement paths of the two heads. The positioning system allows for precise adjustment of the position between the hydrophone and the transducer. The accuracy of displacement and rotation measurement was 0.2 mm and 0.5°, respectively. The data acquisition system is composed of 32 channels, which can be individually used to acquire different signals, both independently and synchronously. The sampling precision is up to 16 bits, the sampling rate is up to 1 MSPS, and the maximum sampling depth is equal to 8192. A PGA can easily realize a 32-channel programmed-change of gain from 0 to 42 dB, with a step of 6 dB.

**Figure 18:**
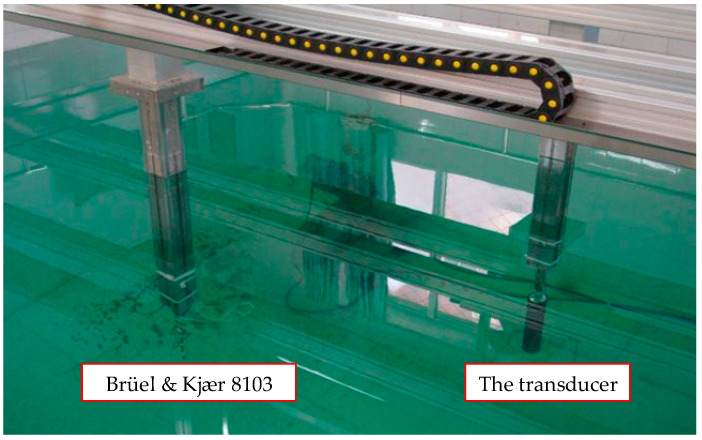
The experimental setup.

**Figure 19:**
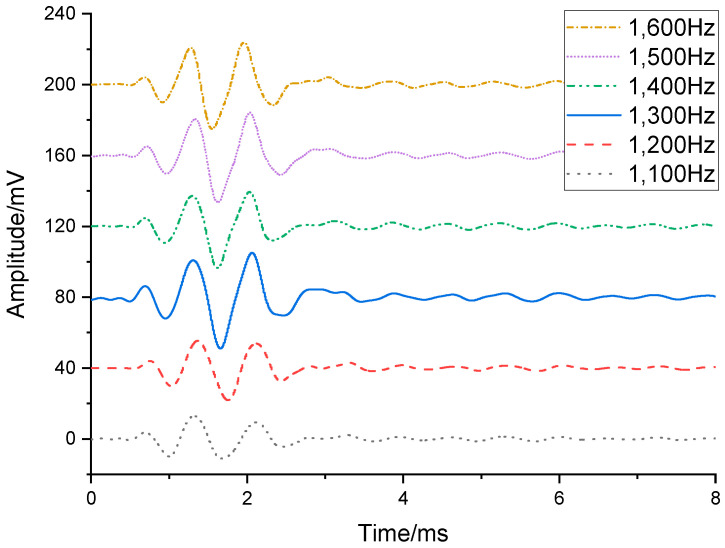
Waveform acquired by hydrophone at 1100–1600 Hz.

**Figure 20:**
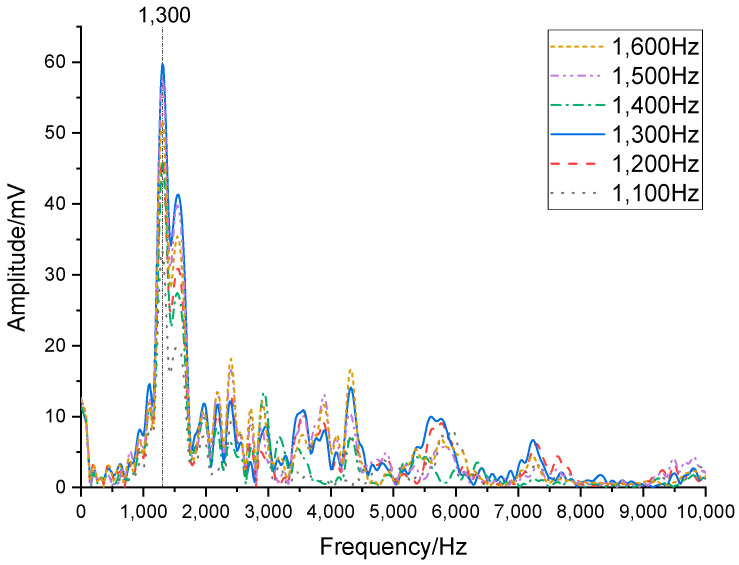
Spectrum diagram of the waveform at 1100–1600 Hz.

In the first phase of the experiment, the transducer and hydrophone were positioned at the center of the water tank, with a separation distance of 1 m between them and a minimum distance of 2 m to the nearest water tank wall. Both the transducer’s flextensional shell and the hydrophone were submerged at 1.6 m depth (2 m clearance from the tank floor. The test setup is shown in Figure 18. Figure 19 displays the hydrophone’s received waveforms under different excitation frequencies applied to the transducer, with the driving frequencies set at 1100 Hz, 1200 Hz, 1300 Hz, 1400 Hz, 1500 Hz, and 1600 Hz. Figure 20 presents the frequency spectrum of this waveform. Results indicate that the transducer exhibited its peak amplitude response at 1300 Hz, which is in close agreement with the simulated result, with a frequency difference of only 38 Hz. It can be confirmed that the resonant frequency of the transducer is 1300 Hz, which offers superior low-frequency capability compared to piezoelectric transducers.

**Figure 21:**
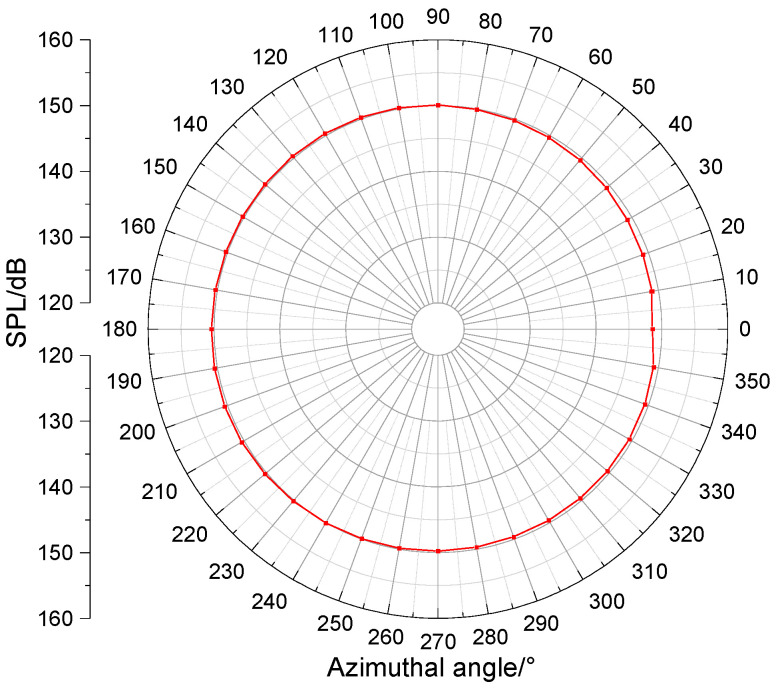
Directivity pattern of the transducer.

In the second phase of the experiment, both the hydrophone and transmitting transducer remained at their original positions in the water tank (identical to the first phase configuration). The transducer was rotated circumferentially, emitting acoustic signals at every 10-degree increment. A complete 360° rotation yielded 36 recorded waveforms via the data acquisition system. The transducer’s acoustic pressure directivity pattern (Figure 21) was then derived through computational analysis of these received waveforms. The average radiated sound pressure level reached 149.8 dB re 1 uPa@1m. The maximum value was 150.5 dB at an azimuth angle of 200 degrees, and the minimum value was 148.5 dB at an azimuth angle of 0 degrees. The acoustic field exhibited good circumferential uniformity. The azimuthal variation was less than 2 dB, which is consistent with the characteristics of a monopole acoustic source.

## 5. Conclusions

An electromagnetic flextensional monopole acoustic logging transducer applicable for borehole environments was developed. Its design employs the Lorentz force as the driving mechanism and a flextensional shell as the acoustic radiator. Finite element simulations were adopted to guide the optimization of the magnetic circuits and flextensional shell, ensuring structural and performance efficiency.

A prototype of the transmitter was fabricated and tested, yielding the following key results: a resonant frequency of 1300 Hz, a sound pressure level (SPL) of approximately 150 dB, and uniform monopole radiation characteristics. Notably, the flextensional shell and driver of the flextensional transducer were physically decoupled—after determining the resonant frequency of the flextensional structure, the operating frequency of the driver circuit can be conveniently tuned to match the mechanical resonance. This feature enables the rapid development of transducers with diverse operating frequencies, as required for different borehole logging scenarios.

In comparison with conventional piezoelectric monopole transducers (which typically achieve 170 dB SPL at higher frequencies), the proposed design exhibited a lower SPL output but offers extended low-frequency capabilities. These low-frequency attributes are critical for two key borehole applications: far-acoustic imaging and Stoneley-wave permeability inversion.

Finally, the experimental results are consistent with the finite element simulation outcomes, verifying the feasibility and practical applicability of the developed transducer for borehole conditions.

In the future, to achieve the mass deployment of this transducer in field applications, further work is required, primarily focused on performance enhancement and field validation. For performance enhancement, the following approaches can be explored: (a) research on higher-performance permanent magnet materials (e.g., advanced NdFeB magnets) and higher-permeability soft magnetic materials to optimize the magnetic circuit structure and increase magnetic flux density, and (b) investigation into incorporating auxiliary coils within the magnetic circuit to further enhance flux density. For field validation, key tasks include: (a) testing the transducer’s adaptability in field logging environments, including high-temperature and high-pressure conditions, and (b) comparative performance evaluation against traditional acoustic sources across various formation conditions to assess signal penetration, stability, and measurement accuracy.

## Data Availability

The data presented in this study are available on request from the corresponding author.

## References

[1] Schmitt D.P., Bouchon M. (1985). Full wave acoustic logging: Synthetic microseismograms and frequency-wavenumber analysis. Geophysics.

[2] Cheng C.H., Toksöz M.N. (1981). Elastic wave propagation in a fluid-filled borehole and synthetic acoustic logs. Geophysics.

[3] Hornby B.E., Johnson D.L., Winkler K.W., Plumb R.A. (1989). Fracture evaluation using reflected Stoneley-wave arrivals. Geophysics.

[4] Sinha B.K., Norris A.N., Chang S.K. (1994). Borehole flexural modes in anisotropic formations. Geophysics.

[5] Sayers C.M., Dasgupta S. (2013). Elastic Anisotropy in the Haynesville Shale from Dipole Sonic Data. Geophys. Prospect..

[6] Chen Y., Zhang G., Wang Z. (2015). Real-time Pore Pressure Prediction Using Machine Learning. SPE.

[7] Li N., Liu P., Wu H.L., Li Y.S., Zhang W.H., Wang K.W., Feng Z., Wang H. (2024). Development and prospect of acoustic reflection imaging logging processing and interpretation method. Pet. Explor. Dev..

[8] Tang X.M. (2004). Imaging near-borehole structure using directional acoustic-wave measurement. Geophysics.

[9] Tang X.M., Patterson D.J. (2009). Single-well S-wave Imaging Using Multicomponent Dipole Acoustic Log Data. Geophysics.

[10] Sinha B.K., Vissapragada B., Renlie L. (2012). Broadband Dipole Sources for Deep Anisotropy Detection. SPE Reserv. Eval. Eng..

[11] Che X., Qiao W., Ju X.D. (2021). Deep Neural Networks for Separating Reflection Waves in Dipole Acoustic Logging. Geophysics.

[12] Wang B., Tao G., Shang X.F. (2017). Dipole Shear-wave Imaging in Salt-bearing Formations. Geophysics.

[13] Che X., Qiao W., Ju X., Wu J., Men B. (2017). Experimental study on the performance of an azimuthal acoustic receiver sonde for a downhole tool. Geophys. Prospect..

[14] Ben J.L., Qiao W.-X., Che X.-H., Ju X.-D., Lu J.-Q., Men B.-Y. (2020). Field validation of imaging an adjacent borehole using scattered P-waves. Pet. Sci..

[15] Ben J.L., Qiao W., Che X., Ju X., Lu J., Men B. (2020). Experimental simulation of obtaining the reflector azimuth using azimuthal acoustic reflection tool in the underwater environment. J. Pet. Sci. Eng..

[16] Cheng L., Che X.-H., Qiao W.-X., Zhao T. (2023). 3D trajectory inversion of an adjacent well using scattered P-wave. Pet. Sci..

[17] Liu P., Fan H.-J., Zhang M.-S., Li Z., Jiang J.-W., Gao Y., Wang K.-W. (2025). Response characteristics of shear waves scattered by fractures with borehole observation system. Pet. Sci..

[18] Li N., Wang K., Liu P., Wu H., Feng Z., Fan H., Smeulders D. (2021). Experimental study on attenuation of Stoneley wave under different fracture factors. Pet. Explor. Dev..

[19] Pan W.G., Feng J., Guan Y. (2018). Evaluation of permeability in medium-porosity and low-permeability formation based on Stoneley wave. J. Appl. Acoust..

[20] Li N., Wang K., Lu J., Liu P., Xiao C., Wu H., Guo Q., Fan H., Men B., Feng Z. (2024). First successful downhole testing of the permeability logging prototype. J. Geophys. Eng..

[21] Li N., Wang K.W., Wu H.L., Feng Q.F., Fan H.J., Smeulders D. (2019). Shock-induced Stoneley waves in carbonate rock samples. Geophysics.

[22] Li N., Wang K.W., Wu H.L., Feng Z., Liu P., Li Y.S. (2023). Permeability logging evaluation: Current status and development directions. Pet. Sci. Bull..

[23] Butler J.L. (1966). Flexural-Extensional Electromechanical Transducer. US Patent.

[24] Rolt K.D. (2005). History of the flextensional transducer. J. Acoust. Soc. Am..

[25] Royster L.H. (1970). The Flextensional Concept: A New Approach to the Design of Underwater Acoustic Transducers. Appl. Acoust..

[26] Folds D.L. (1973). Performance of the Class IV Flextensional Transducer. J. Acoust. Soc. Am..

[27] Royster L.H. (1998). Flextensional Transducers: The Early Years. J. Acoust. Soc. Am..

[28] Lin S., Xu H. (2013). Design of a broadband class IV flextensional transducer with a dual-peak resonance. J. Acoust. Soc. Am..

[29] Butler J.L., Sherman C.H. (2016). Transducers and Arrays for Underwater Sound.

[30] Hu J., Hong L., Yin L., Lan Y., Sun H., Guo R. (2021). Research and Fabrication of Broadband Ring Flextensional Underwater Transducer. Sensors.

[31] Ji Z., Ma Y., Wang Q., Dong C. (2022). Research progress in high-performance soft magnetic alloys. J. Mater. Eng..

[32] Wang T., Zhang S.J., Li F., Liu G. (2020). Thermal Management in High-Power Flextensional Sonar Transducers: ACOMSOL Study. J. Acoust. Soc. Am..

[33] Lin S.Y., Zhang Y. (2018). Multiphysics Simulation of a Broadband Cymbal-Type Flextensional Transducer. IEEE Trans. Ultrason. Ferroelectr. Freq. Control.

[34] Sherrit S., Bao X.Q., Yoseph B.C., Badescu M. (2012). COMSOL Modeling of Flextensional Transducers: Validation with Experimental Data. Proc. SPIE.

